# Associations between Area-Level Unemployment, Body Mass Index, and Risk Factors for Cardiovascular Disease in an Urban Area

**DOI:** 10.3390/ijerph6123082

**Published:** 2009-12-04

**Authors:** Ashley Isaac Naimi, Catherine Paquet, Lise Gauvin, Mark Daniel

**Affiliations:** 1 Department of Epidemiology, Gillings School of Global Public Health, The University of North Carolina at Chapel Hill, Chapel Hill, NC 27599, USA; E-Mail: ashley.naimi@unc.edu; 2 Carolina Population Center, University of North Carolina at Chapel Hill, 104 CPC South, Chapel Hill, NC 27516, USA; 3 Sansom Institute for Health Research, The University of South Australia, Adelaide, SA 5001, Australia; E-Mail: catherine.paquet@unisa.edu.au; 4 Axe santé des populations, CRCHUM (Centre de recherche du Centre hospitalier de l’Université de Montréal), Montréal, Québec H2W 1V1, Canada; E-Mail: mark.daniel@umontreal.ca; 5 Département de médecine sociale et préventive, Université de Montréal, Montréal, Québec H3C 3J7, Canada; E-Mail: lise.gauvin.2@umontreal.ca

**Keywords:** neighbourhood, unemployment, cardiovascular diseases, residence characteristics

## Abstract

**Introduction:**

Cardiovascular Disease (CVD) has been linked to “neighbourhood” socioeconomic status (nSES), often operationalized as a composite index of aggregate income, occupation and education within predefined administrative boundaries. The role of specific, non-composite socioeconomic markers has not been clearly explained. It is also unclear whether the relationship between nSES and CVD varies according to sex. We sought to determine whether area-level unemployment (ALU) was associated with CVD risk, and whether this association differed by sex.

**Methods:**

342 individuals from the Montreal Neighbourhood Survey of Lifestyle and Health provided self-reported behavioural and socioeconomic information. A nurse collected biochemical and anthropometric data. ALU, a weighted average of the proportion of persons 15-years and older available for but without work, was measured using a Geographic Information System for a 250 m buffer centred on individual residence. Generalized Estimating Equations were used to estimate the associations between ALU, body mass index (BMI) and a cumulative score for total cardiometabolic risk (TCR).

**Results:**

After confounder adjustments, the mean 4^th^ minus 1^st^ quartile difference in BMI was 3.19 kg/m^2^ (95% CI: 2.39, 3.99), while the prevalence ratio for the 4^th^ relative to 1^st^ quartile for TCR was 2.20 (95 % CI: 1.53, 3.17). Sex interacted with ALU; women relative to men had greater mean 3.97 kg/m^2^ (95% CI: 2.08, 5.85) BMI and greater mean TCR 1.51 (95% CI: 0.78, 2.90), contrasted at mean ALU.

**Conclusions:**

Area-level unemployment is associated with greater CVD risk, and this association is stronger for women.

## 1. Introduction

Cardiovascular disease (CVD) is an important public health problem [1,2]. Recent research has focused on how social environments shape the distributions of CVD risk factors [3–7] and outcomes [8,9] in a population. In these studies, area-level social deprivation has received much attention. Often gauged by composite indices (usually defined by factor or principal component analysis) using measures of education, income and occupation, there is mounting evidence that area-level social deprivation plays an important role in shaping population rates of CVD [4,9–12].

However, whether the variation observed in a single element of these composite indices is sufficient to elicit a similar association in the relationship between nSES and CVD is less clear. Furthermore, from a policy standpoint, the use of composite social indices can lead to a number of praxis-based challenges insofar as they potentially obscure the independent contributions of each component to specified health outcomes [13,14]. The examination of single variable indictors has utility when policy or public health decision makers may wish to understand the impact of one particular measure on health risk, especially under situations where individual markers may have a high relevance to health risk. In the current economic downturn, area-level unemployment (ALU) may be of unique importance.

Area-level unemployment is a direct measure of urban deprivation [15], and is influenced by policies in fiscal, economic, political, and urban planning domains [16]. High ALU reflects not only income-based deprivation, but also involves issues of gender inequality, social integration, political disenfranchisement and participation, and implicates a lack (or loss) of basic skills and competencies in a given community [16–18]. It is surprising, then, that of numerous studies reporting associations between composite measures of area-level social deprivation and CVD risk factors and events [3,9,12,19–22], only three studies have assessed whether ALU is singularly related to CVD risk factors or events [19,22,23]. Further evidence of such a relationship would provide a tangible point of leverage towards which policy initiatives could be directed, and would be an important complement to recent policy directives aimed at mitigating the impact of the built environment on cardiovascular disease in the population [24].

The most common technique used to analyze area-health associations is to aggregate resident sociodemographic data to administrative group-levels for use in multilevel models. Yet there is a growing awareness of the limitations associated with arbitrarily defined administrative unit measures as ostensibly meaningful neighbourhood constructs [25]. Census tracts and other administrative groupings do not correspond to residents’ perceptions of their neighbourhoods [26], and in contiguous urban areas residents who are closer in space are generally more alike than those farther apart [27]. Arbitrary boundaries that group residents into one or another unit impose distinctions that may not exist in reality [28].

This study examined the associations between ALU and risk factors for cardiovascular disease in a field study of residential area characteristics and individual risk factors for cardiometabolic disease. To represent and ascribe neighbourhood influences we used moving-window areas, corresponding to a perceptually relevant space around the individual, in attempting to reduce misclassification of those residing close to or at the margins of given fixed-boundary [28,29]. We hypothesized that ALU would be associated with elevated BMI and total cardiometabolic risk. Furthermore, given known differences in the determinants of CVD in men and women, we assessed whether associations varied according to sex, after accounting for behavioural, socioeconomic, and area-level covariates.

## 2. Methods

### 2.1. Population and Setting

Data for this study were obtained through the Montreal Neighbourhood Survey of Lifestyle and Health (MNSLH). The Island of Montreal, a densely contained urban centre of 1.8 million residents spread across 521 census tracts (2001 Canada Census data) was the setting for this study. Details have been previously published [30,31]. Briefly, individuals were sampled using a stratified cluster sampling design for seven Census Tracts (CTs) representative of the distribution of CT-level socioeconomic status (nSES) and language groups. Six CTs were initially sampled—three primarily French and three primarily English speaking—across tertiles of an nSES index combining educational attainment and income (one English and one French CT per nSES tertile). A seventh CT was later added to augment low participation in one medium-income French-speaking CT.

Initially, we had sought to recruit 80 individuals per each original CT (480 persons overall). For recruitment of volunteers, informational material was sent to all accessible non-commercial addresses within each CT, followed by a recruiter visit 48 to 72 hours later. A note was left to individuals absent at the first visit inviting them to contact research coordinators if they wished to participate. Contact could not be established with residents of 40% of addresses.

Respondents completed the questionnaire by phone, internet, or on paper. Inclusion criteria were age 18–55 years, no previously diagnosed cardiometabolic disease, and able to read French or English. Eighty percent of individuals reached were eligible, more than the proportion (58.4%) of residents aged between 20–55 years, according to 2001 Canada Census data (11,225/19,225 residents). Of those residents contacted and eligible, 15% agreed to participate. Three-hundred-seventy-four individuals completed the main questionnaire and were contacted for a home visit. Three-hundred-forty-four participants provided additional necessary biological data and two had missing age information, resulting in a final sample size of 342 individuals (71.7% of the original number sought). Participants with missing biological data were mostly from French-language households but did not differ in gender, educational attainment, marital status, income, or fast-food consumption. Compared to Canada Census data for the selected census tracts, 2-sided exact binomial probability tests showed that overall the MNSLH sample over-represented individuals who had a Bachelor’s degree, and those born outside of Canada; higher income and married individuals were over-represented in 3 census tracts.

Questionnaires were completed prior to a home visit at which a registered nurse collected anthropometric measures and finger-prick blood samples during the home-visit. Point-of-care equipment (LDX cholesterol, and GDX hemoglobin A1c analyzers, Cholestech, Hayward, CA) was used to analyze blood samples. All participants gave their informed consent prior to participation. The study protocol was approved by the Human Research Ethics Committee of the Centre de Recherche du Centre Hospitalier de l’Université de Montréal.

### 2.2. Outcome Measures

Finger-prick blood samples were analyzed for glycosylated haemoglobin (% HbA_1c_), triglycerides (TRG; mmol/L), total cholesterol (TC; mmol/L), and high-density lipoprotein cholesterol (HDL; mmol/L). Total cardiometabolic risk (TCR) was estimated as the sum of biological variables above clinical cut-points. Cut-points were based on American Heart Association Guidelines for Primary Prevention of Cardiovascular Disease and Stroke: HbA_1_c ≤ 7.0%; TRG ≤ 1.7 mmol/L; TC ≤ 5.0 mmol/L; HDL ≥ 1.29 mmol/L for women and 1.03 mmol/L for men [32]. Body Mass Index (BMI) was calculated as weight (kg)/height (m^2^) and analyzed in continuous form. BMI and TCR were analyzed separately to ascertain whether area-level effects might be differentially associated with anthropometric vs. haematologic CVD antecedents.

### 2.3. Exposure Measures and Covariates

#### 2.3.1. Area Level Measures

Area-level socioeconomic and sociodemographic information was obtained from 2001 Canada Census data incorporated into a comprehensive Geographic Information System [33]. Moving-window areas representing immediate “neighbourhood” influences [28] were created by geo-linking census-level data to a 250 m radius buffer centred on an individual’s residential address, using GeoPinpoint^©^ Software (DMTI Spatial).

The exposure measure, ALU, was determined from the census-based unemployment rate, defined as the percentage of individuals “15 years and over, excluding institutional residents, who, during the week (Sunday to Saturday) prior to Census Day, were without paid work or without self-employed work and were available for work and either: (a) had actively looked for paid work in the past four weeks; (b) were on temporary lay-off and expected to return to their job; (c) had definite arrangements to start a new job in four weeks or less.” [34]. Using this definition, ALU was calculated for resident-centred 250 m buffers. A weighted average of the unemployment rate was calculated for CTs over which the buffer overlapped, with weights corresponding to overlap area. The same technique was used to represent area-level education as the proportion of the population 20 years and older with at least a grade 9 education. In order to increase the discriminative ability of ALU but maintain parsimony, we chose *a priori* to categorize ALU into quartiles (Range: Q1 = 4.51%–8.81%; Q2 = 8.86%–10.62%; Q3 = 10.62%–14.44%; Q4 = 15.20%–20.80%), and area-level education into tertiles (Range: Q1 = 1.29%–9.46%; Q2 = 9.49%–14.64%; Q3 = 14.71%–27.48%).

#### 2.3.2. Individual Level Measures

Individual-level covariates considered were physical activity, and consumption of fruits and vegetables, fast food and alcohol, in addition to education, income, and employment status. Potential confounders were age, smoking status, and area-level education (specified using a Directed Acyclic Graph, details available on request from first author).

*Physical activity* was assessed via questionnaire inquiring about overall time spent walking, time spent walking *specifically* for health, and time spent in vigorous physical activity over the previous week. This information was converted to the number of Metabolic Equivalents (METS; a measure of energy expenditure as multiples of resting metabolic rate) expended over the previous week and operationalized as a standard score. *Fruit and vegetable consumption* was assessed using a modified version of the U.S. Behavioral Risk Factor Surveillance System questionnaire [35,36]. Consumption of eight different groupings of fruits and vegetables over the previous week, ranging from “None” to “Every day,” was self-reported. A total fruit and vegetable consumption score was calculated based on the sum of responses to the eight five-point items and operationalized as a continuous variable. *Fast food consumption* was estimated using a proxy measure of the number of fast food restaurant (FFR) visits in the previous week, self-reported on a four-point scale ranging from 0–5 times or more per week. This score was dichotomized using a cut-off of one or more FFR visits in the previous week, based on a clear inflection in the variable’s distribution. A score of zero was used as referent. *Alcohol consumption* was measured by a question on the quantity of alcohol consumed over the previous week. Responses were categorized as “abstainer,” “light drinker” (women ≤ 1 drink/day; men ≤ 2 drinks/day) and “heavy drinker” (women > 1 drink/day; men > 2 drinks/day), based on 2005 USDA/HHS Dietary Guidelines [37]. “Abstainer” was used as the referent. Finally, *smoking status* was self-reported and categorized as smoker/non-smoker, with non-smoker as referent.

Education and income were assessed using two 9-point scales requiring respondents to indicate the highest level of education completed and total yearly household income, respectively. Education was operationalized as a dichotomous variable with greater than or equal to a high-school education as referent. Income was operationalized using two dummy variables for total yearly household income between $CAD 20,000 and $CAD 50,000, and $CAD 50,000 plus. Employment status was determined via questionnaire and operationalized as a dichotomous variable. Unemployed status was used as the referent. Demographic covariates included age (categorized as a continuous variable) and gender (male as referent).

### 2.4. Statistical Analysis

Analyses were conducted using SPSS 14 [38]. Generalized Estimating Equations (GEE) with an exchangeable correlation matrix were used to simultaneously estimate the effects of area- and individual-level predictors on BMI and TCR outcomes while accounting for clustering of respondents within CTs [39,40]. Associations with the continuous BMI measure were estimated for a normal distribution with an identity link function. A Poisson regression model (log link) was used to estimate prevalence ratio (PR) associations with TCR (a count measure). After confirming that ALU was associated with TCR, we conducted a *post hoc* analysis with each TCR sub-component using the binomial distribution model (logit link function), with results expressed as odds ratios (OR).

Four regression models were fitted to assess the relationships between ALU and outcomes, with covariates introduced in blocks. Models 1 and 2 included DAG-defined confounders and serve as primary inferential models. Model 1 included individual-level confounders (age and smoking status), while Model 2 included the Model 1 covariate block as well as area-level education. Models 3 and 4 were specified in order to render our parameter estimates comparable to studies that adjust for intermediary variables. Sex-specific associations were calculated, running all four models within sex strata. The magnitude and confidence limits of differential associations (presented in the Abstract and Section 3.2.3) were derived from an interaction term added to Model 2.

Model diagnostics included Pearson residuals plotted against the predicted value of the Linear Predictor [41]. Four outliers were observed. Since results did not differ between models including and excluding outliers, analyses were performed with complete data. Assessment of Variance Inflation Factors (VIFs) indicated no multicollinearity among the predictor variables (VIF Range = 1.08–1.77).

## 3. Results

### 3.1. Descriptive Statistics

Table 1 presents the behavioural, socioeconomic and biological characteristics of the study participants according to sex.

In general, women had similar BMIs but a more favourable TCR profile relative to men. Relative to women, men exercised more, frequented fast food establishments and were unemployed more often, smoked more, and consumed more alcohol.

### 3.2 Associations between ALU, BMI, and TCR

Table 2 presents relationships between ALU and BMI, and ALU and TCR for statistical Models 1 through 4.

A gradated relationship was apparent across ALU quartiles for both BMI and TCR. This relationship was unchanged after accounting for area- and individual-level covariates.

#### 3.2.1. Body Mass Index

There was a monotonic, positive association between BMI and ALU. Relative to the first quartile, the magnitude of association increased slightly upon adjusting for area-level education, and decreased slightly upon inclusion of individual education, income and employment status (Model 3) and behavioural covariates (Model 4).

#### 3.2.2. Total Cardiometabolic Risk

Similar to BMI analyses, there was a monotonic, positive association between TCR and ALU, even after adjusting for covariates. For quartiles 2–4, associations were unchanged upon the inclusion of age, area-level education, and markers of individual socioeconomic status, relative to the referent (first) quartile. Sub-component analysis revealed an increase in the magnitude of the association after adjustment for the three series of covariates (Models 2 to 4) in all components except Total Cholesterol (results not shown). Furthermore, as Table 3 demonstrates, in Model 4, the association was strongest for HbA_1c_, followed by TRG, HDL, and TC.

#### 3.2.3. Gender Stratified Analysis

Gender specific models revealed differences in the magnitude of association for both BMI and TCR models (Table 4). Model 2 interaction terms revealed that, for women the 4th to 1st ALU quartile difference in BMI was 3.97 kg/m^2^ (95% CI: 2.08, 5.85), greater than the difference for men in the 4th to 1st ALU quartile. Similarly, for Model 2, the ratio of TCR prevalence ratios for women in the 4th relative to the 1st ALU quartile was 1.51 (95% CI: 0.78, 2.90) times as high as the ratio for men in the 4th relative to the 1st ALU quartile.

## 4. Discussion

In our sample of urban residents in seven census tracts, area-level unemployment was positively associated with body mass index, and a cardiometabolic risk score representing the number of elevated risk factors for cardiometabolic disease. These associations held even after adjusting for area-level education, individual-level education, income and unemployment status, fruit and vegetable, fast food, alcohol, tobacco consumption and physical activity. Furthermore, women had stronger associations than men in associations between ALU, BMI and TCR.

Our findings are consistent with two of the total of three published studies that assessed area-level unemployment in relation to CVD. These studies, carried out in (i) a combined German and Czech [19] and (ii) Swedish [23] cohorts, documented relationships between area-level unemployment and obesity [19], and first hospitalization for a fatal or nonfatal coronary heart disease event [23]. Unlike the present report, neither of these studies accounted for behavioural variables in estimating measures of association, thus limiting their comparability to many published research studies. In addition, one study [19] looked only at individuals aged 45–69, omitting those most vulnerable to CVD events associated with BMI [40].

The third study, based in Montreal, assessed the association between BMI and community unemployment operationalized at the level of police districts for a sample of n = 2043 individuals, finding no association [22]. However, Montreal police districts (n = 49) are large administrative units containing a mean of 36,700 residents, compared to CTs (n = 521) with a mean of 3,500 residents. Furthermore, BMI calculated from self-reported height and weight was used to categorically operationalize respondents as obese or non-obese. Categorical estimates of BMI based on self-reported height and weight are prone to misclassification [43], which could partly explain why no association was observed beyond the possibility that the large administrative groupings with underlying heterogeneity masked associations that might otherwise have been apparent.

Molinari *et al*. [11] and Ellaway and Macintyre [44] have suggested that relationships between the social environment and health outcomes are likely to differ between men and women. Molinari *et al*. [11] reported that, for perceived health, women are more likely than men to be affected by perceptions of the social environment. Our findings provide support for the notion that the social environment may be more strongly associated with the health status of women than with men, as our measures were more objective representations of health and social context. We cannot rule out however a potential influence of the built, in addition to social, environment. Such attributes are likely to be related in a given locale, and the degree to which a given constituent can be differentiated is not straightforward [45]. Future research should investigate this question in more detail, especially with regards to whether social and built environmental factors relate differently to the health of men and women.

Although we adjusted for a broad spectrum of covariates, strong associations remained. This may be due to unmeasured factors that influence the effects of area-level unemployment on BMI and total cardiometabolic risk, such as psychosocial status—measures of which are implicated as potential mediators of area-health relationships [25,46]. Alternatively, part of the association might reflect a direct link between the social environment and the individual, in which non-conscious cognitions influence one’s allostatic and cardiometabolic status [25]. Additional research is required to evaluate potential causal mechanisms through which area effects are expressed.

This study has limitations worth noting. The cross-sectional design precludes causal inference; our limited sample size limits point estimate precision; and self-selection of participants introduces potential bias. The most problematic source of potential bias in our study is the limited response rate, which would suggest that our sample might not be representative of the source population. To further investigate this, we conducted an ancillary analysis comparing the proportions of 18 sociodemographic measures (representing dimensions of age, education, language, household size, income, unemployment, marital status, and immigrant status) in our study sample to the actual proportions in the 7 CTs from which our sample was derived. Of the 18 measures, our sample differed from the source population only with respect to age (7 of the 7 CTs), marital status (4 of the 7 CTs), immigrant status (4 of the 7 CTs), and education (6 of the 7 CTs). Furthermore, the differences observed were minor, with a mean (SD) difference in proportion of 0.11 (0.08) for marital status, 0.26 (0.07) for age, 0.21 (0.11) for education, and 0.09 (0.06) for immigrant status (first generation). The two largest differences we observed (age and education) were to be expected, given our inclusion criterion for respondents aged 18–55 years, and the tendency for individuals with higher levels of education to participate more willingly in epidemiological studies [47]. With respect to the two smaller differences (marital and immigrant status), it is known that first-generation immigrants are more likely to be leaner than their non-immigrant counterparts [48], and that married individuals have better cardiovascular profiles than non-married individuals [49]. Thus, if either status played an important biasing role in our study, the effect would most likely have been towards the null. Our results are unlikely to reflect over controlling, since the nature of the associations evaluated remained consistent as new covariates were added to our models. An additional issue is neighbourhood scale. We used a 250 m buffer zone to represent immediate “neighbourhood” influences, but the utility of scales has not yet been resolved in studies of area effects, and it is possible that other radii may be more or less appropriate. Finally, endogeneity [50] was not considered; our protocol did not ask whether residents resided where they did for health reasons.

In summary, area-level unemployment within the proximal 250 m area of individual residence is associated with higher BMI and greater total cardiometabolic risk, even accounting for key area- and individual-level covariates. The observed associations were greater for women than for men. The basis of these differential relationships requires further investigation, preferably by longitudinal design.

## Figures and Tables

**Table 1.** Sample characteristics of neighbourhood study participants (n = 342).

	**Men (n = 169)**	**Women (n = 173)**

**Continuous Variables**	**Mean (Std Dev)**	**Mean (Std Dev)**
BMI (kg/m^2^)	25.1 (3.9)	24.6 (5.2)
Age (years)	35.8 (8.9)	33.9 (8.5)
Weekly energy expenditure (METS)	1348.6 (1052.2)	1063.8 (856.5)
Fruit & Vegetable Consumption (Max = 40)	13.2 (4.9)	14.2 (4.1)

**Categorical Variables**	**N (%)**	**N (%)**
Unemployed		
Yes	27 (16.0)	13 (7.5)
No	142 (84.0)	160 (92.5)
Area-Level Unemployment		
Quartile 4	33 (19.5)	43 (24.9)
Quartile 3	45 (26.6)	48 (27.7)
Quartile 2	47 (27.8)	48 (27.7)
Quartile 1	44 (26.0)	34 (19.7)

Fast Food Consumption		
Yes	87 (51.5)	61 (35.3)
No	82 (48.5)	112 (64.7)

Smoker		
Never smoker/former smoker	113 (66.9)	125 (72.3)
Smoker	56 (33.1)	48 (27.7)

Education		
Less than high school	9 (5.3)	18 (10.4)
High-School completed	35 (20.7)	26 (15.0)
Trade school or university	125 (74.0)	129 (74.6)

Alcohol Consumption		
Abstainer	55 (32.5)	64 (37.0)
Moderate	80 (47.3)	97 (56.1)
Heavy	33 (19.5)	11 (6.4)

Income		
Below $20K (CAD)	44 (26.0)	57 (32.9)
Between $20K & 50K (CAD)	61 (36.1)	52 (30.1)
Above $50K (CAD)	64 (37.9)	64 (37.0)

Total Cardiovascular Risk		
0 no indicator exceeding risk value	39 (22.8)	62 (35.8)
1 indicator exceeding risk value	51 (29.8)	73 (42.2)
2 indicators exceeding risk value	44 (25.7)	28 (16.2)
3 indicators exceeding risk value	28 (16.4)	9 (5.2)
4 indicators exceeding risk value	7 (4.1)	1 (0.6)

**Table 2.** Associations between area-level unemployment, body mass index (BMI) and total cardiometabolic risk (TCR) (n = 342).

		**Model 1†**	**Model 2a‡**	**Model 3§**	**Model 4#**
		**Beta (95% CI)**	**Beta (95% CI)**	**Beta (95% CI)**	**Beta (95% CI)**
BMI	ALU4*	2.69 (2.40, 3.00)	3.19 (2.39, 3.99)	2.71 (1.93, 3.49)	2.11 (1.03, 3.19)
	ALU3	1.67 (1.12, 2.22)	2.16 (1.71, 2.61)	1.71 (1.14, 2.78)	1.51 (0.55, 2.47)
	ALU2	0.50 (0.11, 0.90)	1.56 (0.46, 2.66)	1.37 (0.59, 2.15)	1.09 (−0.20, 2.38)

		**PR (95% CI)**	**PR (95% CI)**	**PR (95% CI)**	**PR (95% CI)**

TCR	ALU4*	1.60 (1.47, 1.73)	2.20 (1.53, 3.17)	1.85 (1.32, 2.59)	1.82 (1.35, 2.44)
	ALU3	1.50 (1.36, 1.65)	1.84 (1.44, 2.33)	1.60 (1.25, 2.04)	1.66 (1.33, 2.07)
	ALU2	1.16 (1.07, 1.25)	1.42 (0.99, 2.03)	1.28 (0.92, 1.77)	1.37 (0.97, 1.94)

* Referent is first (lowest) quartile throughout. GEEs were used for all models with a Normal distribution (identity link function) for BMI and a Poisson distribution (log link function) for TCR.

† Model 1 included age, gender, and smoking status.

‡ Model 2 included age, gender, smoking status, and area-level education.

§ Model 3 included age, gender, smoking status, area-level education, and individual education, income and employment status.

# Model 4 included age, gender, smoking status, area-level education, individual education, income and employment status, physical activity, fast-food consumption, fruit and vegetable consumption and alcohol consumption.

**Table 3.** Odds Ratios for Total Cardiometabolic Risk Score Sub-Component Analysis.*

		**HDL (95% CI)**	**TRG (95% CI)**	**TC (95% CI)**	**HbA_1c_ (95% CI)**
Model 1†	ALU4	2.72 (2.40, 3.08)	2.52 (2.12, 2.97)	1.04 (0.62, 1.72)	1.82 (1.65, 2.01)
	ALU3	2.09 (1.31, 3.32)	1.96 (1.67, 2.3)	0.765 (0.40, 1.46)	2.07 (1.88, 2.27)
	ALU2	0.73 (0.58, 0.91)	0.83 (0.71, 0.95)	1.346 (0.80, 2.24)	1.98 (1.73, 2.25)
Model 2‡	ALU4	5.93 (2.07, 16.95)	4.93 (1.64, 14.81)	1.465 (0.68, 3.12)	6.32 (3.61, 11.04)
	ALU3	4.14 (1.30, 13.15)	1.97 (1.04, 3.72)	0.997 (0.58, 1.7)	2.64 (1.78, 3.89)
	ALU2	0.93 (0.76, 1.12)	0.98 (0.71, 1.34)	1.592 (1.01, 2.53)	2.74 (2.33, 3.21)
Model 3§	ALU4	4.85 (1.77, 13.24)	4.33 (1.38, 13.50)	0.948 (0.44, 2.00)	6.13 (2.53, 14.79)
	ALU3	3.83 (1.33, 10.96)	1.93 (1.12, 3.30)	0.791 (0.49, 1.27)	2.62 (1.54, 4.42)
	ALU2	0.95 (0.83, 1.07)	1.05 (0.75, 1.44)	1.45 (0.98, 2.13)	2.64 (2.12, 3.26)
Model 4#	ALU4	4.19 (1.18, 14.84)	4.51 (1.05, 19.24)	0.987 (0.46, 2.09)	7.45 (3.78, 14.68)
	ALU3	2.68 (0.82, 8.71)	1.82 (0.94, 3.52)	0.778 (0.51, 1.18)	2.68 (1.55, 4.61)
	ALU2	0.61 (0.46, 0.79)	0.99 (0.50, 1.92)	1.404 (1.25, 1.57)	2.85 (2.19, 3.71)

* Referent is first (lowest) quartile throughout. GEEs with a binomial distribution (logit link function) were used for all models.

† Model 1 included age, gender, and smoking status.

‡ Model 2 included age, gender, smoking status, and area-level education.

§ Model 3 included age, gender, smoking status, area-level education, and individual education, income and employment status.

# Model 4 included age, gender, smoking status, area-level education, individual education, income and employment status, physical activity, fast-food consumption, fruit and vegetable consumption and alcohol consumption.

**Table 4.** Association between area-level unemployment (ALU), body mass index (BMI) and total cardiometabolic risk (TCR) for 169 men and 173 women.

		**BMI**		**TCR**	
		**Men**	**Women**	**Men**	**Women**
		**Beta (95% CI)**	**Beta (95% CI)**	**PR (95% CI)**	**PR (95% CI)**
Model 1^†^	ALU4^*^	0.8 (0.33, 1.27)	4.63 (3.94, 5.32)	1.36 (1.02, 1.81)	2.1 (1.49, 2.95)
	ALU3	−0.32 (−1.26, 0.62)	3.65 (2.87, 4.43)	1.37 (1.02, 1.83)	1.58 (1.08, 2.31)
	ALU2	−1.7 (−2.27, −1.13)	2.53 (1.86, 3.20)	1.20 (0.88, 1.67)	1.13 (0.76, 1.69)
Model 2^‡^	ALU4	0.96 (−0.96, 2.88)	5.7 (1.96, 9.44)	1.85 (1.26, 2.72)	3.00 (1.10, 8.19)
	ALU3	−0.53 (−1.73, 0.67)	4.5 (1.93, 7.07)	1.56 (1.16, 2.11)	2.09 (0.83, 5.25)
	ALU2	−0.14 (−2.02, 1.74)	3.08 (0.96, 5.20)	1.25 (0.77, 2.04)	1.46 (0.68, 3.12)
Model 3^§^	ALU4	1.45 (−0.82, 3.72)	4.89 (0.83, 8.95)	1.64 (1.13, 2.39)	2.38 (0.98, 5.79)
	ALU3	0.18 (−1.2, 1.55)	3.89 (1.26, 6.52)	1.42 (1.03, 1.96)	2.64 (0.67, 4.02)
	ALU2	0.04 (−1.78, 1.86)	3.18 (0.87, 5.49)	1.19 (0.71, 2.01)	1.27 (0.61, 2.64)
Model 4^#^	ALU4	1.69 (−0.47, 3.85)	2.7 (−1.44, 6.85)	1.61 (1.19, 2.18)	2.51 (1.12, 5.6)
	ALU3	0.57 (−0.80, 1.94)	2.25 (−1.06, 5.56)	1.47 (1.18, 1.84)	1.82 (0.77, 4.28)
	ALU2	0.18 (−2.19, 2.55)	1.71 (−1.37, 4.79)	1.26 (0.82, 1.94)	1.41 (0.74, 2.7)

* Referent is first (lowest) quartile (ALU1) throughout. GEEs were used for all models with a Normal distribution (identity link function) for BMI and a Poisson distribution (log link function) for TCR.

† Model 1 included age and smoking status

‡ Model 2 included age, smoking status, and area-level education

§ Model 3 included age, smoking status, area-level education, and individual income, education and employment status.

# Model 4 included age, smoking status, area-level education, individual income, education and employment status, fresh fruit and vegetable consumption, fast food consumption, physical activity and alcohol consumption.
